# Cellular Responses to the Efferocytosis of Apoptotic Cells

**DOI:** 10.3389/fimmu.2021.631714

**Published:** 2021-04-20

**Authors:** Charles Yin, Bryan Heit

**Affiliations:** ^1^ Department of Microbiology and Immunology, Schulich School of Medicine and Dentistry, Western University, London, ON, Canada; ^2^ Center for Human Immunology, Western University, London, ON, Canada; ^3^ Robarts Research Institute, London, ON, Canada

**Keywords:** efferocytosis, intracellular trafficking, transcriptional regulation, cellular metabolism, inflammation resolution, host defense

## Abstract

The rapid and efficient phagocytic clearance of apoptotic cells, termed efferocytosis, is a critical mechanism in the maintenance of tissue homeostasis. Removal of apoptotic cells through efferocytosis prevents secondary necrosis and the resultant inflammation caused by the release of intracellular contents. The importance of efferocytosis in homeostasis is underscored by the large number of inflammatory and autoimmune disorders, including atherosclerosis and systemic lupus erythematosus, that are characterized by defective apoptotic cell clearance. Although mechanistically similar to the phagocytic clearance of pathogens, efferocytosis differs from phagocytosis in that it is immunologically silent and induces a tissue repair response. Efferocytes face unique challenges resulting from the internalization of apoptotic cells, including degradation of the apoptotic cell, dealing with the extra metabolic load imposed by the processing of apoptotic cell contents, and the coordination of an anti-inflammatory, pro-tissue repair response. This review will discuss recent advances in our understanding of the cellular response to apoptotic cell uptake, including trafficking of apoptotic cell cargo and antigen presentation, signaling and transcriptional events initiated by efferocytosis, the coordination of an anti-inflammatory response and tissue repair, unique cellular metabolic responses and the role of efferocytosis in host defense. A better understanding of how efferocytic cells respond to apoptotic cell uptake will be critical in unraveling the complex connections between apoptotic cell removal and inflammation resolution and maintenance of tissue homeostasis.

## Introduction

Efferocytosis is the process of rapid and efficient clearance of apoptotic cells by both professional and non-professional phagocytic cells (1, 2). From an evolutionary perspective, efferocytosis is an ancient mechanism that allowed early multicellular organisms to regulate their growth through the disposal of dying cells during development (3). In complex multicellular organisms, efferocytosis is critical in growth and development, for the resolution of inflammation, and for maintaining tissue homeostasis (4–6). Mechanistically, efferocytosis closely resembles phagocytosis—the internalization and clearance of pathogens and other foreign particulates (7). Indeed, though efferocytosis utilizes a distinct and well-characterized set of cell surface receptors (e.g. TAM family receptors, Tim4, α_V_ integrins) and soluble opsonins (e.g. Gas6, MFGE8, CD93) that bind to ligands found on the plasma membrane of apoptotic cells (e.g. phosphatidylserine), much of the processes downstream of apoptotic cell internalization such as intracellular trafficking of apoptotic cell cargo and cellular responses to internalized of apoptotic cell contents are either thought to be wholly analogous to phagocytosis or to be poorly understood (1, 7–10). Strikingly, efferocytes such as macrophages can distinguish between normal apoptotic cells and those infected with an intracellular pathogen, despite the fact that much of the contents of an infected apoptotic cell (e.g. lipids, nucleic acids, proteins) are identical to that of a non-infected cell, allowing the efferocyte to mount an appropriate immunological response to pathogens within efferocytosed cells (3). This demonstrates that efferocytosis is a distinct process from phagocytosis, that and efferocytes are fine-tuned to be able to distinguish between apoptotic versus pathogenic cargo.

The major efferocytic cell—or efferocyte—within the body is the macrophage (11). These immune cells are responsible for clearance of apoptotic cells and debris across many tissues (12, 13). There is emerging evidence that efferocytic macrophages form a distinct subset of tissue-resident macrophages that differ in both function and pattern of gene expression compared to other tissue-resident macrophage populations (14, 15). Indeed, A-Gonzalez et al. (14) found that tissue-resident murine efferocytic macrophages from across a range of different tissues share a common transcriptional profile, which is characterized by downregulation of proinflammatory cytokines such as IL1β and expression of the mannose receptor CD206 (14). Interestingly, although this population upregulates several anti-inflammatory genes, it’s gene expression profile does not co-cluster with alternatively activated (M2) macrophages (14). This suggests that efferocytic macrophages cannot simply to be thought of as “anti-inflammatory” macrophages, and instead occupy a distinct space in the macrophage transcriptional landscape.

The purpose of this review will be to discuss the distinct cellular responses elicited upon uptake of apoptotic cells by an efferocyte, with a focus on macrophages as the major efferocytic cell population within the body. In particular, we will review differences in trafficking of apoptotic cell cargo and presentation of antigens following internalization, alterations in cell signaling and transcriptional regulation, as well as explore how changes in cargo trafficking and gene expression contribute to the anti-inflammatory phenotype that characterize efferocytes. Further, we will explore recent advances in our understanding of how efferocytes deal with the metabolic stress of internalizing apoptotic cells, how efferocytes respond upon uptake of infected apoptotic cells, and the role of efferocytosis in host defense.

## Efferosome Trafficking and Antigen Presentation

**Figure 1:**
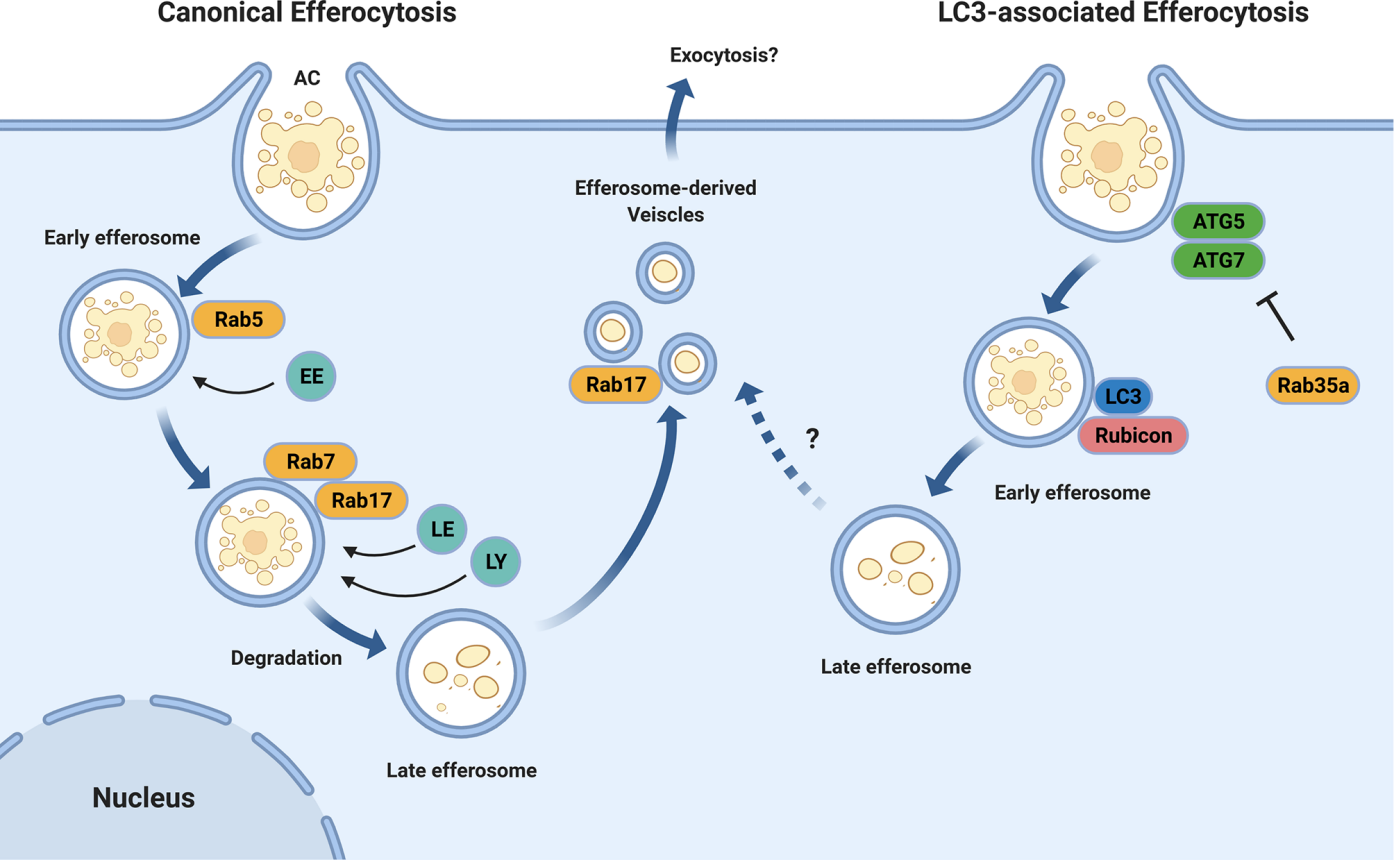
Efferosome Maturation Pathways. Efferocytosis can occur through the canonical endo-lysosomal maturation pathway (left) in which the GTPases Rab5 and Rab7 mediate the sequential fusion of early endosomes (EE), late endosomes (LE), and lysosomes (LY) with the maturing efferosome. Unlike phagocytosis, this efferosome maturation pathway also involves Rab17 which directs the degraded contents from the efferosome to the recycling endosome from where they may be exocytosed, thereby avoiding the delivery of these materials to antigen loading compartments. In addition to the canonical pathway, some efferosomes may mature through an LC3-mediated, autophagy-like pathway (right). In this pathway, the efferosome recruits the protein LC3 which then mediates a rapid degradation of the efferosome in a fashion which suppresses antigen presentation. Similar to LC3-associated phagocytosis, the recruitment of the autophagy-related proteins ATG5 and ATG7, as well as Rubicon to the nascent efferosome appear to be important for efferocytosis through this pathway. The activity of ATG5 and ATG7 are inhibited by Rab35a, which is activated downstream of TLR signaling. Figure produced using BioRender.

Following recognition, apoptotic cells are engulfed by the efferocyte into a plasma membrane-derived vacuole termed an efferosome (8). Similar to phagosomes that contain internalized pathogens, efferosomes undergo a highly regulated series of sequential fusions with early endosomes, late endosomes, and finally lysosomes (**Figure 1**) (16–18). These fusion events are regulated by proteins including Rab GTPases and SNAREs, with the fusion events delivering the hydrolytic enzymes which degrade the apoptotic cell within the efferosome (19–23). This process is termed efferosome maturation and is analogous to the maturation processes observed following phagocytosis and endocytosis (8, 24).

The efferosome maturation process bears many similarities to phagosome maturation, including the recruitment of the Rab GTPases Rab5 and Rab7 (23, 25, 26). Rab5 is recruited to efferosomes as the apoptotic cell is internalized, and remains bound to the efferosome for several minutes following the release of the efferosome from the plasma membrane (19, 27). Here, Rab5 mediates the fusion of the efferosome with early endosomes, beginning the degradative process which will ultimately disassemble the apoptotic cell (19, 20). Rab5 is exchanged for Rab7 several minutes after efferosome formation, with Rab7 mediating the fusion of late endosomes and lysosomes to the efferosome – thus generating a highly hydrolytic environment capable of the complete degradation of the apoptotic cell (18, 27). Recent work by our group and others have demonstrate important differences between the regulation of efferosome maturation versus phagosome maturation (27, 28). Efferosome acidification is a central process that facilitates the degradation of apoptotic cargo through activation of lysosomal proteases (29, 30).

Efferosomes have also been shown to employ LC3-associated phagocytosis (LAP, **Figure 1**), a noncanonical form of autophagy that involves recruitment of autophagy mediators including the class III phosphatidylinositol-3-kinase (PI3KCIII) complex ATG5 and ATG7 to the surface of nascent efferosomes (31, 32). These elements then direct the rapid maturation of the efferosome and processing of the apoptotic cargo in a manner that suppresses antigen presentation and serves to polarize macrophages towards an anti-inflammatory phenotype (33). While the exact signals which allow for LAP to be employed for efferosome maturation remain unknown, work from the Medzhitov group has demonstrated that phagosome-derived Toll-like receptor (TLR) signaling is required to direct materials into the classical phagocytic (e.g. non-LAP) pathway where they then undergo antigen presentation (34, 35). This indicates that the detection of pathogen products *via* TLR’s serves not only to induce the expression of genes involved in inflammation and antigen presentation, but also induces immediate differences in the trafficking of cargo bearing TLR ligands compared to those lacking these ligands (36). Rab39a may serve to inhibit LAP following phagocytosis, as this GTPase inhibits autophagy following TLR signaling, and is required for the delivery of MHC I to phagosomes for antigen cross-presentation (37, 38). However, there are no published studies of the role of Rab39a in efferocytosis, and therefore its role in efferocytosis-associated LAP remains unclear. Interestingly, LAP and the formation of LC3-associated efferosomes is dependent on the Beclin1-interacting protein Rubicon (39). Rubicon is a negative regulator of canonical autophagy and downregulation of this protein results in an increase in the number of autophagosomes (39, 40). Indeed, deletion of Rubicon in a mouse model of autoimmune disease significantly increases susceptibility to the development of systemic lupus erythematosus-like features in these animals, potentially due to altered processing of apoptotic cells (41).

Differences in acidification and trafficking of efferosomes, as compared to phagosomes, also plays a role in ensuring the immunologically silent degradation of apoptotic cells (34, 42, 43). There is conflicting evidence in the literature on the kinetics of efferosome maturation as compared to phagosome maturation (35, 42). Erwig et al. reported that in murine macrophages, early maturation and acidification of efferosomes containing apoptotic neutrophils proceeded at a faster rate than phagosomes containing IgG-opsonized neutrophils (42). Inhibition of the small GTPase RhoA using a small molecule inhibitor was sufficient to negate these differences (42). In contrast, Blander and Medzhitov have shown that efferosome maturation proceeded at a slower rate than phagosomes (35). Of note, in the case of Blander and Medzhitov, the phagocytic target employed was inactivated *Escherichia coli* and the authors argue that it was activation of TLR2 and TLR4 signaling that drove accelerated phagosome maturation (35). In contrast, the IgG-coated neutrophils used by Erwig and colleagues would not have stimulated TLRs in the same fashion (42).

Our group has recently demonstrated that efferosome localization appears to play a role in distinguishing the fate of apoptotic cargo (28). Canonically, phagosomes undergo dynein-mediated trafficking towards the cell centre as they mature, where lysosomes are concentrated due to a similar dynein-mediated trafficking pathway (16, 44–46). Thus, by moving to the cell centre, phagosomes can efficiently undergo fusion with lysosomes to acquire the hydrolytic enzymes that degrade phagosome cargos (29, 47). In contrast, we have shown that while efferosomes also undergo an initial migration towards the cell centre where they fuse with lysosomes, they subsequently fragment into smaller efferosome-derived vesicles (EDVs) which migrate away from the cell centre and towards the periphery (28). At the periphery, EDVs undergo fusion with the recycling endosome compartment, presumably to facilitate exocytosis of degraded apoptotic cargo or resorption of nutrients (28). This process is driven by the small GTPase Rab17 (**Figure 1**), which is required for both the fragmentation of efferosomes into EDVs and for the movement of the EDVs to the cell periphery (27, 28). Macrophages that overexpress a dominant-negative mutant of Rab17 accumulate efferosomes at the cell center (28). Furthermore, the presence of Rab17 on efferosomes also prevents the delivery of MHC class II, circumventing autoantigen presentation from degraded apoptotic cargo (27). Expression of a dominant-negative Rab17 impairs this pathway, leading to MHC II accumulation in mature efferosomes (27).

The presence of three processes that work simultaneously to limit antigen presentation of efferosome-derived antigens – LAP, accelerated maturation, and Rab17-mediated redirection of cargo out of the maturing efferosome – indicates that limiting autoantigen presentation is a fundamental response of phagocytes following efferocytosis. Moreover, efferocytes engage in non-trafficking-based mechanisms to limit autoimmune responses to efferocytosed materials. As described later in this review, efferocytosis is often accompanied by the upregulation of cytokines such as IL-10 which suppress the activity of mature T cells and promotes the formation of T_reg_ cells from naive T cells (48). Consequentially, T cell responses are inhibited following efferocytosis. For example, Rodriguez-Fernandez et al. demonstrated that in human DCs, efferocytosis of PtdSer-containing liposomes biased the stimulation of autologous T cells from a proliferative to a tolerogenic profile, likely through altered cytokine expression by the DCs (49). Consistent with these mechanisms acting to limit autoreactivity, emerging evidence indicate that defects in the suppression of antigen presentation following efferocytosis is a driver of autoimmune disease (50, 51). In mouse models of systemic lupus erythematosus, dysregulated expression of specific pro-efferocytic receptors such as Tim4, C1q or CLM-1 result in either deficient apoptotic cell clearance or inappropriate antigen presentation that then promotes the development of autoimmune disease in these mice (52). In humans, mutations in efferocytic receptors, especially in MERTK and its opsonins, are associated with a similar increase in the risk of autoimmune disorders including multiple sclerosis and rheumatoid arthritis, highlighting the importance of efficient efferocytosis in limiting autoimmunity (53–57).

Interestingly, some professional antigen presenting cells have mechanisms that allow efferosome-derived antigens to be cross-presented on MHC I. A recent study by Canton et al. demonstrated that type 1 conventional dendritic cells use the receptor DNGR-1 to recognize actin-myosin complexes exposed to the efferosome lumen during the early stage of efferosome maturation (58). Recognition of actin-myosin complexes leads to an alternative maturation pathway where the efferosome does not acquire its normal degradative capacity, and instead, Syk-induced NADPH oxidase activity damages the efferosomal membrane, releasing the efferosome’s cargo into the cytosol. Once in the cytosol, the efferocytosed materials are processed and presented *via* the canonical MHC I presentation pathway [reviewed in (59)]. Interestingly, the restriction of this process to the early stages of efferosome maturation suggests that this process may only occur in response to engulfed cells that have pre-exposed actin-myosin complexes – e.g. cells which have lost membrane integrity as they progress through late stage apoptosis, or cells which have died a lytic form of cell death such as necroptosis or necrosis (9). Alternatively, this pathway may enable the routine “screening” of apoptotic cell-derived antigens *via* MHC I, which because it relies on T cells previously activated to the same antigen presented on MHC II by professional antigen presenting cells, lacks the autoimmune potential of MHC II presentation (60).

## Cell Signaling and Transcriptional Regulation

Differences between the cellular response of phagocytes to efferocytosis of apoptotic cells versus phagocytosis of pathogens require that there be efferocytosis-specific signal transduction events and transcriptional regulation (1, 2). We are just beginning to develop an understanding of the key transcriptional factors that control the cellular events that occur following efferocytosis. Two key families of transcriptional factors that drive this response are members of the liver X receptor (LXR) and peroxisome proliferator-activated receptor (PPAR) families of nuclear receptors (61–63). These transcription factor families bind to the same DNA motifs, and often act as heterodimers, meaning that their functions are often overlapping and redundant (64). Both receptor families bind to many of the same ligands, notably lipid-derived metabolites, with their activation leading to the preferential formation of heterodimers that then bind to direct 5’ – RGKTCA – 3’ repeats (65). Once bound, these LXRs and PPARs coordinate with other transcription factors to either activate or repress transcription (64, 66, 67).

LXRs are well-characterized regulators of cholesterol, glucose and fatty acid metabolism (68). The two members of the LXR family, LXRα and LXRβ, are both activated following efferocytic apoptotic cell uptake, and in turn increase the cell’s efferocytic capacity *via* two distinct mechanisms (69). The first mechanism – described in-detail later in this review – is the upregulation of the metabolic pathways required to process the large quantities of lipids, sterols and proteins present in an efferocytosed apoptotic cell. The second mechanism is the upregulation of efferocytic receptors and signaling molecules. Stimulation of LXRs *in vivo* with apoptotic thymocytes has been shown to upregulate MERTK, a key efferocytic receptor involved in apoptotic cell recognition and uptake (69, 70). This enhances the efferocytic capacity of the efferocyte, and increases MERTK-mediated anti-inflammatory activity *via* increased activation of SOCS3, a suppressor of cytokine-induced JAK/STAT signaling (71). Conversely, peritoneal macrophages isolated from LXR double-knockout mice have been shown to have diminished capacity to engage in efferocytosis, without any impairment in the phagocytosis of *E. coli* (69). Indeed, activation of LXRα/β appears to be required to shift macrophages away from a pro-inflammatory state following efferocytosis, with exposure of LXR double-knockout macrophages to apoptotic thymocytes resulting in increased expression of several pro-inflammatory mediators including IL1β, MCP-1 and the scavenger receptor MARCO (69).

Given the functional overlap between LXRs and PPARs, it is of no surprise that the observed role of the PPAR family in efferocytosis closely parallels the role of LXRs. As with LXRs, PPARs have previously been implicated in macrophage polarization and in enhancing lipids metabolism and synthesis of lipid-derived molecules such as eicosanoids and arachidonic acid (72). Similar to the LXR family of transcription factors, activation of certain members of the PPAR family, including PPARγ and PPARδ, appear to directly enhance efferocytic activity in macrophages (63, 66, 73). Majai et al. demonstrated that downregulation of PPARγ activity using a small-molecule inhibitor resulted in a diminished capacity of human monocyte-derived macrophages to efferocytosed apoptotic neutrophils (66). This resulted from the downregulation of several key efferocytic receptors including CD36, AXL, TG2 and PTX3 (66). Using PPARγ-specific agonists, Zizzo & Cohen demonstrated that PPARγ activation leads directly to upregulation of MERTK and its opsonin Gas6 in macrophages, as well as to polarization of macrophages to a pro-efferocytic M2c phenotype (74). Furthermore, efferocytosis of apoptotic cells by macrophages has been shown to directly suppress key inflammatory pathways, including activation of PKCα, a kinase involved in many cellular functions including inflammatory cytokine transcription and the generation of bactericidal free radicals (64). Indeed, activation of PPARγ in response to efferocytosis of apoptotic cells in murine macrophages has been shown to attenuate reactive oxygen species formation in response to proinflammatory mediators (64). Similarly, efferocytosis induces the expression of SOCS1 and SOCS3, which in turn inhibit Jak/STAT signaling through inflammatory cytokine receptors, thereby reducing the responsiveness of efferocytic macrophages to inflammatory stimuli (75). Finally, the uptake of apoptotic thymocytes by murine bone marrow-derived macrophages has been shown by Mukundan and colleagues to upregulate PPARδ and stimulate PPARδ-dependent expression of C1qb, a member of the complement cascade that has been identified as an opsonin involved in the efferocytic clearance of apoptotic macrophages (63).

Defects in efferocytosis have been implicated in the pathogenesis of several inflammatory and autoimmune disorders, including atherosclerosis (76, 77). Our group recently discovered that atherosclerotic macrophages upregulated the hematopoietic transcription factor GATA2 in response to modified lipoproteins (78). Upregulation of GATA2 led to the downregulation of multiple proteins required for efficient efferocytosis, including downregulation of the efferocytic receptor α_X_ integrin, multiple signaling molecules required for these receptors function including multiple Src-family kinases, impaired efferosome-lysosome fusion *via* decreased expression of Rab7, and impairment in multiple degradative pathways needed for the degradation of apoptotic cargos including lysosomal acidification (10, 78). Interestingly, mutations in the GATA2 gene has been linked to increased risk of cardiovascular disease in human cohort studies (79). It remains to be seen whether there are other transcription factors that act to impair efferocytosis during autoimmune or inflammatory diseases.

## Resolution of Inflammation

A key feature of efferocytosis is the limitation of inflammation and the resolution of inflammatory responses (5, 9). We have previously discussed how efferosome maturation acts to prevent antigen presentation on MHC II, and how efferocytosis activates transcriptional programs that restrain inflammation (27, 78). It is well established that efferocytosis induces the production of anti-inflammatory mediators (80, 81). Meagher et al. showed as early as 1992 that the uptake of apoptotic neutrophils by macrophages does not lead to release of the pro-inflammatory mediator thromboxane A2, in contrast with phagocytosis of bacterial pathogens (80). Only a few years later Fadok and colleagues demonstrated that efferocytosis in macrophages resulted in suppression of a host of proinflammatory molecules including IL1β, IL8, IL10, GM-CSF and TNFα (81). Furthermore, these investigators determined that efferocytosis upregulated anti-inflammatory mediators including TGFβ and prostaglandin E2 (81).

More recent studies have demonstrated that efferocytic macrophages carry anti-inflammatory functions and gene expression signatures. A landmark study in 2017 showed that pro-efferocytic macrophages across various tissues carried a distinct gene expression signature that differentiated them from other tissue-resident macrophages (14). In particular, this pro-efferocytic signature is characterized by downregulation of the inflammatory cytokine IL1β (14). Campana et al. further demonstrated that in a sterile liver inflammation model, efferocytosis of apoptotic hepatocytes induced a M2-like phenotype and activation of the STAT3-IL6-IL10 pathway (82). Finally, in an acute coronary ligature model, Howangyin and colleagues demonstrated that mouse macrophages lacking the efferocytic receptor MERTK and its opsonin MFGE8 had decreased production of the vascular tissue repair factor VEGF-A and increased tissue damage in a model of myocardial infarct (83).

Beyond simply downregulating the production of pro-inflammatory factors, there is growing evidence that efferocytosis also directly induces the resolution of inflammation (61, 84). Specialized pro-resolving mediators (SPMs) are a class of signaling molecules including resolvins and lipoxins that are derived from free fatty acids that play a key role in limiting inflammation in physiological settings (85). The work of Cai et al. demonstrated that mice lacking the efferocytic receptor MERTK have decreased levels of LXA_4_ and RvD1 when challenged with zymosan in a model of inducible peritonitis (86). These authors further demonstrated that activation of MERTK using a cross-linking antibody resulted in decreased levels of the enzyme 5-lipooxygenase in the macrophage nucleus, which has previously been shown to result in increased SPM production (86). Interestingly, SPM signaling enhances the efferocytic capacity of macrophages and reduces their sensitivity to efferocytosis-induced cell stress, suggesting that SPM production may be a self-reinforcing stimuli which acts in an autocrine or paracrine manner to enhance the efferocytic capacity within a tissue when apoptotic cells are present (87, 88).

## Efferocyte Metabolism

**Figure 2:**
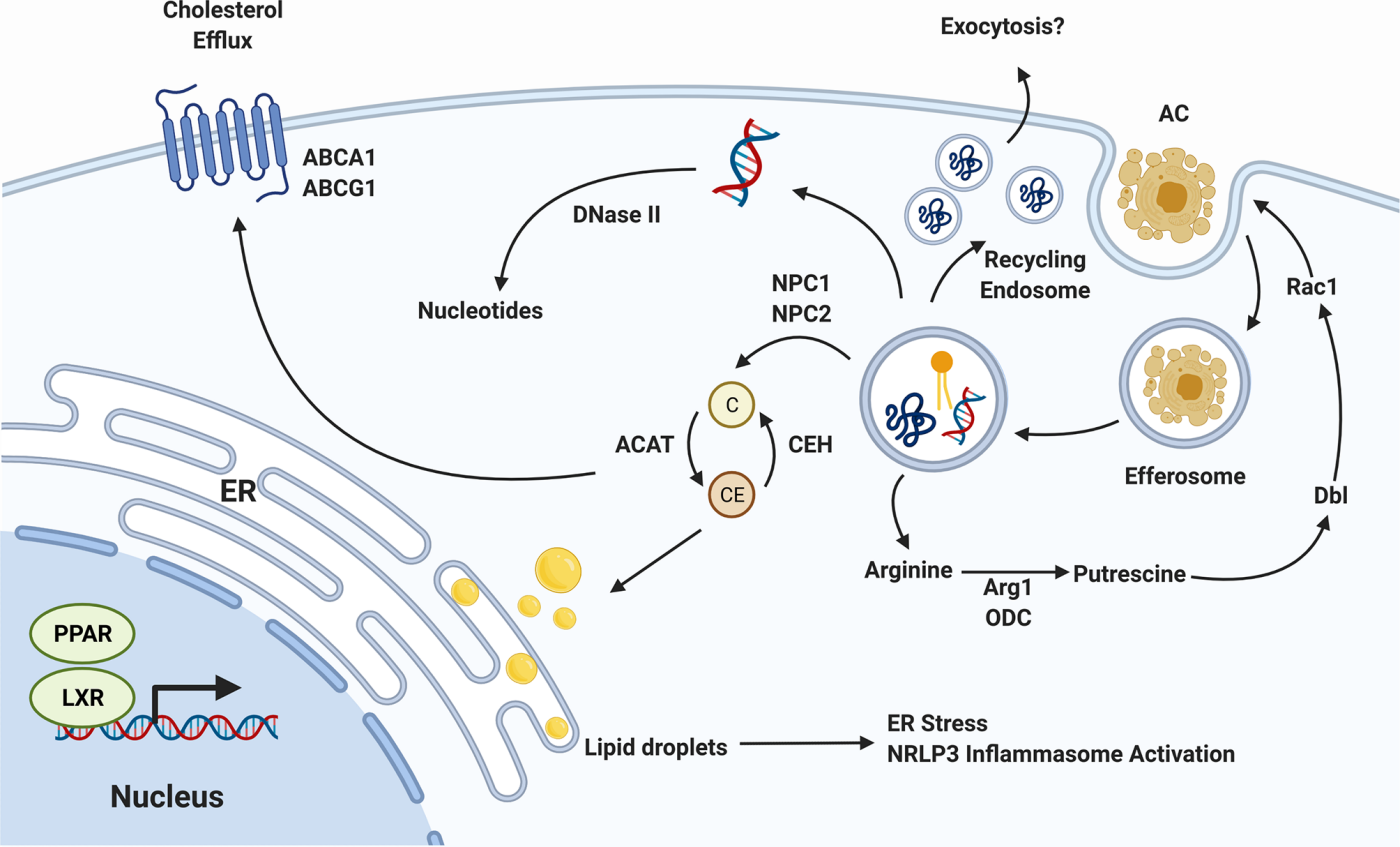
Efferocyte Metabolism. The biomolecules released as efferocytosed apoptotic cells are degraded must be processed by the efferocyte, incurring a significant metabolic load. Cholesterol (C) is exported from the efferosome to cytosolic carriers which, in the presence of cholesterol transporters such as pABCA1 and ABCG1, can export this cholesterol to circulating high density lipoprotein. In the absence of sufficient export, cholesterol is esterified into cholesterol esters (CE) which can accumulate in the endoplasmic reticulum (ER). DNA is degraded in the efferosome by DNase II, and proteins by a range of cathepsin and other proteases, with the resulting nucleotides and amino acids transported into the cytosol where they are recycled. The amino acid arginine is converted in the cytosol to the putrescine, which activates Dbl to enhance Rac1 activity, thereby promoting the efferocytosis of additional apoptotic cells. Lastly, the activation or PPAR and LXR nuclear receptors by lipid-derived metabolites induces a pro-efferocytic metabolic profile *via* upregulation of cholesterol export machinery and upregulation of lipid β-oxidation. Figure prepared in BioRender.

The uptake and degradation of apoptotic cells places a unique metabolic demand on efferocytes (89). These cells must not only quickly degrade the apoptotic cell, but must also ensure that components of the degraded apoptotic cell - especially excess lipids and cholesterol - are redistributed and not allowed to accumulate within the efferocyte (90, 91). A failure to prevent the accumulation of metabolites such as cholesterol and lipids is a source of significant cellular stress that promotes inflammation and can lead to the death of the efferocyte (92, 93). Evidence indicates that efferocytes such as macrophages have unique means of dealing with this additional metabolic load (94, 95). Lipid catabolism is enhanced *via* a distinctive metabolome characterized by an increase in the generation of ATP from the β-oxidation, accompanied by a concordant enhancement of the mitochondrial electron transport chain, fatty acid oxidation, and oxidative phosphorylation (**Figure 2**) (94). These adaptations allow efferocytes to rapidly process excess lipids obtained from internalized apoptotic cells.

Efferocytes have multiple molecular mechanisms in place to deal with the metabolic stress induced by cholesterol accumulation, most of which converge on increasing the rate of cholesterol export from the cell (96, 97). Following uptake of an apoptotic cell, cholesterol is exported from the efferosome by NPC1 and NPC2 to cytosolic cholesterol carriers (98). These carriers transport cholesterol throughout the cell, but in the absence of cholesterol export, these carriers ultimately deliver cholesterol to the ER (98, 99). Here, cholesterol accumulates within the ER membrane, eventually forming lipid droplets (100). Unaddressed, these droplets can accumulate to the point where they induce the ER’s unfolded protein response, leading to apoptosis of the efferocyte (92). Efferocytes such as macrophages increase the expression of genes involved in cholesterol export to avoid this fate, notably the cholesterol efflux pumps ABCA1 and ABCG1, which export cytosolic cholesterol to lipid-poor apolipoproteins and HDL (**Figure 2**) (101, 102). Macrophages have multiple pathways by which these cholesterol efflux pumps can be induced. This includes the induction of ABCA1 transcription by LXR following apoptotic cell uptake (103). In parallel, signaling through the efferocytic receptor BAI1 induces a signaling through the BAI1/ELMO/Rac1 pathway that leads to upregulation of ABCA1 in an LXR-independent manner (104). Both the LXR-dependent and -independent pathways enable macrophages to export excess cholesterol absorbed during efferocytosis, thus maintaining cholesterol homeostasis within the cell and avoiding death of the efferocyte (103, 104). The consequences of impaired cholesterol efflux can be dire. The increased ER stress caused by lipid droplet formation not only leads to death *via* the unfolded protein response but is also inflammatory due to activation of the NLRP3 inflammasome (105). In addition to causing cell death, the accumulation of cholesterol can directly impair efferocytosis. In one study, Viaud et al. inhibited lysosomal acid lipase, an enzyme required for hydrolysis of cholesterol esters within lysosomes into free cholesterol prior to their export *via* NPC1/2 to cytosolic carriers (106). This resulted in accumulation of cholesterol esters within the lysosome interfered with Rac1 activation, blocking the engulfment of additional apoptotic cells (106).

In addition to excess lipids and cholesterol, efferocytes must also deal with excess amino acids, short peptides, and apoptotic cell DNA (107). While amino acids and peptides are exported from efferosomes by lysosomal transporters, and via trafficking to the recycling endosome, apoptotic cell DNA is degraded by DNase II in professional efferocytes such as macrophages (108). This is a critical step in maintaining the immunologically silent nature of efferocytosis, with deletion of DNase II from macrophages resulting in the upregulation of pro-inflammatory mediators such as TNFα, likely *via* activation of TLR9 by partially digested DNA fragments containing unmethylated CpG motifs (108, 109).

Another important alteration to cellular metabolism following efferocytosis are those allowing for additional rounds of efferocytosis (110). Professional efferocytes such as macrophages must often clear multiple apoptotic cells in succession, and impaired clearance of multiple apoptotic cells is regarded as a marker of defective efferocytosis (110, 111). Several components of cellular metabolism are altered in order to facilitate continuous efferocytosis. Wang et al. showed that efferocytic uptake of apoptotic cells induced Drp1-mediated mitochondrial fission along with mitochondrial calcium ion release (110). When this fission process was inhibited macrophages lost their ability to successively engulf apoptotic cells. These macrophages exhibited defective sealing of the efferosome and decreased continuous efferocytic capacity (110). Interestingly, this process is accompanied by a loss of mitochondrial membrane potential driven by the uncoupling protein Ucp2, increased glucose uptake *via* SLC2A1, and a shift to glycolysis over oxidative phsophroylation (2, 111, 112). In parallel, these cells upregulate the lactate transporter SLC16A1, enabling the rapid export of the end-product of glycolysis (112). This shift in cellular energetics may be required to sustain rapid, successive apoptotic cell uptake and degradation, although how the decoupling of oxidative phosphorylation observed in these studies occurs in cells seemingly also requiring increased oxidative phosphorylation for the β-oxidation of fatty acids remains unresolved (94, 95, 112). Broadly speaking, mitochondrial fission and fusion are important processes that serve to regulate mitochondrial DNA segregation, mitochondrial reactive oxygen species levels and calcium homeostasis (113). These processes have also been shown to be coupled to particular metabolic states in macrophages (113, 114). For example, classically activated, pro-inflammatory macrophages require massive upregulation of glycolysis within the cell (114). Nair et al. demonstrated that blockade of mitochondrial fission with Mdivi-1, a mitochondrial division inhibitor, led to reversal of metabolic reprogramming towards glycolysis in macrophages treated with LPS (115). Therefore, alteration of mitochondrial fusion and fission following efferocytosis may represent alignment with the unique metabolic state adopted by efferocytes following apoptotic cell internalization. Finally, recent work has shown that apoptotic cell-derived arginine and ornithine are converted by macrophages into to putrescine through the activity of the enzymes arginase 1 and ornithine decarboxylase (116). Putrescine subsequently increases Rac1 activity through upregulation of the GTP exchange factor Dbl, enhancing the ability of the efferocyte to engulf additional apoptotic cells (**Figure 2**) (116). During efferocytosis, macrophages further process putrescine into other polyamines such as spermidine and spermine, but these don’t appear to have the same efferocytosis-enhancing effect as putrescine (116). However, it should be noted that some polyamines, in particular spermidine, confer protection from atherosclerosis by promoting enhanced cholesterol efflux and appear to have cardioprotective effects in animal models of heart failure (117, 118).

## Host Defense

An often-underappreciated role of efferocytosis is its role in host defense (3, 119). Efferocytosis plays an important role in control of intracellular pathogens, most notably, control of *Mycobacterium tuberculosis* (120). In its natural life cycle, *M. tuberculosis* is internalized by macrophages, where it persists within the phagosome by halting phagosome maturation prior to acidification (121). But while *M. tuberculosis* can proliferate within these phagosomes, the infected macrophages eventually undergo apoptosis and are cleared through efferocytosis by other, healthy macrophages (3, 121). Because the bacterium is trapped within the apoptotic cell, it is unable to inhibit efferosome maturation as efficiently as it inhibits phagosome maturation (122). Consequentially, the clearance of infected macrophages by efferocytosis is an important mechanism for controlling *M. tuberculosis* through killing within fully matured efferosomes (3, 120). Importantly, the efferocytic degradation of *M. tuberculosis*-infected apoptotic cells results in antigen presentation on MHC II – unlike what is observed with uninfected apoptotic cells. While the exact mechanism which allows for the normally non-immunogenic efferocytic pathway to result in antigen presentation remains unclear, it is mediated at least in part by annexin 1, which is required for cross-presentation of *M. tuberculosis* antigens on MHC I to CD8+ T cells (123).

The ability of macrophages to recognize intracellular pathogens within infected apoptotic cells is a relatively new finding, and the mechanisms that underlie this process remain incompletely defined. It is thought that engagement of TLRs within the maturing efferosome is required, with TLR4 known to be required for the recognition of infected apoptotic cells in other models (124). The detection of pathogens within apoptotic cells is not restricted to bacteria. Efferocytosis of apoptotic cells infected by the herpes simplex virus appears to trigger recognition within the efferosome and subsequent preparation and cross-presentation of viral antigens to CD8+ T cells, where it plays an important role in the control of the virus in a mouse models of infection (125).

It is also unclear whether differences exist in how efferocytes handle the processing of excess cholesterol, lipids and nucleic acids derived from apoptotic cell uptake should that cell be infected with an intracellular pathogen. Indeed, the lipidomic response to pathogen phagocytosis appears to be the opposite of that following efferocytosis. Lipid synthesis is increased following pathogen phagocytosis, including synthesis of ceramides on the phagosome itself (126), and an accompanying upregulation of lipogenesis *via* TLR-mediated activation of the transcription factors sterol regulatory element binding transcription factor 1 and 2 (SREBP1/2) (127–130). To our knowledge however, no study to date has examined whether a similar phenomenon occurs in maturing efferosomes or whether there is any difference in how efferocytes handle excess lipids and other metabolites following uptake of an infected apoptotic cell.

## Discussion

Efferocytosis is an essential homeostatic mechanism which clears apoptotic cells and debris before the dying cell progresses to necrosis and induces an inflammatory response (1, 13). Although mechanistically similar to phagocytosis, efferocytosis is mediated by a distinct set of receptors, engages a unique maturation pathway, and ultimately results in the efficient degradation of internalized apoptotic cells while avoiding antigen presentation and inflammation (16). To engage in efferocytosis, macrophages take on a unique gene expression and metabolic profile to ensure they are equipped with the necessary metabolic capacity to process the contents of multiple dying cells (14, 94). In this review, we explored several cellular responses to apoptotic cell uptake observed in efferocytes, especially in professional efferocytic cells such as macrophages.

The process of efferosome maturation is similar to that of phagosome maturation, with processes ultimately resulting in cargo degradation (8). However, while phagosomes acquire antigen-presentation machinery – resulting in the presentation of phagosome-derived antigens on both MHC I and II – efferosomes avoid this process and instead dispose of apoptotic cargo in an immunologically silent fashion (1, 28). Similarly, while phagocytosis results in activation of several signaling cascades that lead to generation of a pro-inflammatory response, efferocytosis engages a different set of pathways which upregulate anti-inflammatory and tissue remodeling mediators. This is accomplished through the activation of distinct transcription factors in cells undergoing phagocytosis versus efferocytosis. Indeed, efferocytic macrophages carry a common gene expression signature associated with these functions that distinguish them from pro-inflammatory, tissue patrolling, and other tissue-resident macrophages (14). Efferocytosis of apoptotic cells also appears to induce a unique set of metabolic adaptations designed to permit the efferocyte to effectively deal with the increase burden of lipids, cholesterol and other apoptotic cell-derived macromolecules, while simultaneously priming the cell to engage in additional rounds of efferocytosis (94, 110). Finally, efferocytosis has a role in host defense against intracellular pathogens, including both bacteria and viruses (119, 120, 125).

Although there have been significant advances in our understanding of efferocytosis over the past few decades, there remain significant gaps in our understanding – especially regarding the role of efferocytosis in pathogen clearance, and in our understanding of the metabolic reprogramming of efferocytes. In particular, we current lack a detailed mechanistic understanding of how efferocytes are able to distinguish between infected versus non-infected apoptotic cells. It is further unclear how efferocytes respond metabolically to infected apoptotic cells. Furthermore, it remains unclear whether differences in immunological outcomes following pathogen versus apoptotic cell uptake are solely the result of differences in the receptors used to recognize each type of cargo, or if the processing of cargo within the phagosome or efferosome also plays a significant role. Finally, we are only beginning to unravel the complexities of immunometabolic responses to apoptotic cell uptake and further work is needed to fully define how efferocytes are able to cope efficiently with the massive intake of lipids, proteins and nucleic acids. With a growing body of evidence that defects in efferocytosis are involved in inflammatory and autoimmune disease, a clearer understanding of how professional efferocytes such as macrophages respond to apoptotic cell uptake will be crucial in furthering our understanding of the pathogenesis of these disorders and identifying potential therapeutic options.

## Author Contributions

CY and BH contributed equally to the authoring of this manuscript. All authors contributed to the article and approved the submitted version.

## Funding

This study was funded by a Heart and Stroke Foundation of Canada Grant-In-Aid and an Ontario Ministry of Research and Innovation Early Research Award to BH. CY was funded by a Vanier PhD Scholarship and Canadian Institutes for Health Research (CIHR) MD/PhD studentship.

## Conflict of Interest

The authors declare that the research was conducted in the absence of any commercial or financial relationships that could be construed as a potential conflict of interest.

## Abbreviations

LAP, LC3-associated phagocytosis; PI3KCIII, class III phosphatidylinositol-3-kinase; TLR, Toll-like receptor; DCs, dendritic cells; PtdSer, phosphatidylserine; LXR, liver X receptor; PPAR, peroxisome proliferator-activated receptor; SPM, pro-resolving mediator.
